# Targeting Appropriate Interventions to Minimize Deterioration of Drinking-water Quality in Developing Countries

**Published:** 2008-06

**Authors:** Andrew F. Trevett, Richard C. Carter

**Affiliations:** ^1^Environmental Health Unit, World Health Organization, GPO Box 250, Dhaka 1000, Bangladesh; ^2^School of Applied Sciences, Cranfield University, UK

**Keywords:** Diarrhoea, Disease risk index, Drinking-water, Household, Hygiene, Interventions, Review literature

## Abstract

In developing countries, it has been observed that drinking-water frequently becomes recontaminated following its collection and during storage in the home. This paper proposes a semi-quantified ‘disease risk index' (DRI) designed to identify communities or households that are ‘most at risk' from consuming recontaminated drinking-water. A brief review of appropriate physical and educational intervention measures is presented, and their effective use is discussed. It is concluded that incorporating a simple appraisal tool, such as the proposed DRI, into a community water-supply programme would be useful in shaping the overall strategy requiring only a minimum of organizational learning.

## INTRODUCTION

Conservative estimates indicate that around 1.5 billion people worldwide use an engineered water supply, such as a public standpipe or community well which requires collection and storage of water in the home (1). Although the quality of water supplied by such systems may be up to the standard of the World Health Organization (WHO) guideline at the point of supply (2), it has been well-documented that deterioration between collection and consumption is a widespread though not a universal problem (3). In other words, drinking-water that is of acceptable quality at the point of supply becomes microbiologically contaminated during the distinct processes of collection, transportation, and household storage. In this paper, the terms—‘recontaminated' or ‘recontamination'—will be used for refering to such deterioration of drinking-water.

The severity of the health risk from consuming recontaminated drinking-water will vary among communities, households, and individuals. This is because water-handling practices, other related factors, and decisively the health and immunity status of the individual together determine the health risk. It would be useful if the ‘most-at-risk' communities or households could be identified to design appropriate interventions. For this purpose, a rapid appraisal tool is needed which could be used by extension workers in community water-supply programmes. The information provided by such a tool should form the basis of an intervention strategy that is tailored to the community context.

Recontaminated drinking-water undermines the positive health impacts of providing improved water supply. There is, therefore, a need to employ appropriate intervention measures that will prevent or minimize deterioration of water quality. These may include physical interventions, such as specially-designed water-storage containers, and educational campaigns that promote good hygiene behaviour. A carefully-planned and implemented intervention strategy may well be successful in maintaining safe drinking-water up to the point of consumption. Nevertheless, it is important to be realistic about what can be achieved in terms of reducing diarrhoeal disease in communities that face a multitude of poverty-related problems.

This paper proposes a semi-quantified ‘disease risk index' (DRI) that is designed to identify communities or households that are ‘most at risk' from consuming recontaminated drinking-water. The DRI is a rapid appraisal tool whose primary purpose is to collect information for use in the planning of an intervention strategy. The paper also reviews several intervention measures which are intended to minimize deterioration of water quality and considers how they can be most effectively used. A literature review of physical and educational interventions was undertaken using established databases, including the Web of Science® and ScienceDirect. This was supplemented with information sourced from Internet searches of websites of international agencies involved in treatment of household water and promotion of hygiene. Finally, key issues with regard to developing an intervention strategy are discussed in the context of community water-supply programmes. This section considers the rationale for tailored interventions and examines the value of the DRI in designing interventions more successfully.

## BACKGROUND

**Fig. 1:**
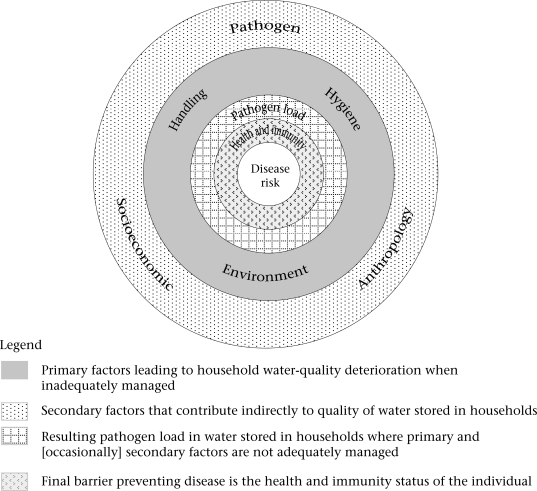
A conceptual framework showing the primary and secondary factors that determine the potential pathogen load in drinking-water stored in households and the final barrier preventing disease

In a previous paper, we presented a conceptual framework that describes how several interrelated factors affect the potential pathogen load in drinking-water stored in households (4). Figure 1 illustrates the conceptual framework, and Table 1 briefly defines the primary factors—‘handling', ‘hygiene', ‘environment', and secondary factors—‘pathogen', ‘anthropology' and ‘socioeconomics'. Where stored drinking-water contains sufficient numbers of a pathogen to constitute an infective dose, the final barrier preventing disease is the status of the health and immunity of the individual.

**Table 1:** Definition of primary and secondary factors in the conceptual framework used for describing their interaction to affect the pathogen load in drinking-water stored in households

Factor	Definition
Handling	Practices surrounding collection, storage, and serving of drinking-water
Hygiene	Refers specifically to hand-washing in this context
Environment	Sanitary quality of the household and community environment
Pathogen	Nature of pathogens, e.g. persistence, virulence, infective dose
Anthropology	Cultural values and norms of the community (lifestyle factors)
Socioeconomic	Levels of education (especially hygiene) and income

Primary factors are those which can be tackled by micro-level interventions, namely physical and educational interventions. Of the secondary factors, only the ‘socioeconomic' factor can be addressed by intervention, and practically, this would focus at macro-levels. Additionally, an understanding of cultural aspects of community life—the ‘anthropological' factor—is important to ensure that interventions are appropriate and sustainable.

### Disease risk index

Indices of disease risk linking household faecal contamination or family hygiene with diarrhoeal morbidity in children have been validated in field studies (5,6). The value of these indices is in identifying specific behaviours or practices that are associated with diarrhoeal disease and predicting the households most at risk. Ideally, such indices should be designed for use as part of a rapid appraisal toolkit.

We propose a DRI that is specific to the health risks from consuming recontaminated drinking-water. The basic premise is that an assessment of factors that determine the potential pathogen load in household drinking-water, combined with an assessment of the health and immunity of household members, can be used for predicting the level of health risk. The DRI is derived by carrying out a rapid appraisal of these two components, hereafter referred to as ‘household water quality', and ‘health and immunity'. The appraisals are assigned a score or classification which is then used for determining the DRI.

Results of our research in rural Honduran communities revealed how the primary and secondary factors indicated in the conceptual framework (Fig. 1) interact and can result in the substantial recontamination of drinking-water (1,4). The understanding gained from this research forms the basis of the ‘household water quality' appraisal. Each of the primary and secondary factors, with the exception of the ‘pathogen' factor, is set out in the household water-quality score sheet (Table 2). The ‘pathogen' factor is excluded because simple pathogen-identification techniques do not exist at present, and in any case, all diarrhoeal pathogens represent a health risk to infants.

**Table 2:** Household water-quality score sheet. The observation guide is used for determining scores for the primary and secondary factors. The total score is used in conjunction with an assessment of health and immunity to derive the disease risk index which indicates the health risk from consuming recontaminated drinking-water

Primary and secondary factors	Score	Observation guide used for differentiating between factor scores
Environment: ‘Sanitary quality of the household and community environment'	2	None or very few latrines; livestock and poultry regularly seen inside the house; human faeces visible in and around the house; floors not swept
	1	Partial latrine coverage; efforts to keep animals out of house but chicks reared inside; animal faeces present in and around house but human faeces rarely seen
	0	Latrine coverage greater than 65%; no human or animal faeces seen in or around house; animals fenced in; floors swept; eating/drinking utensils stored
Hygiene: ‘Refers specifically to hand-washing in this context'	2	Hand-washing rarely observed; hands evidently soiled; nails blackened; absence of hand-washing facilities
	1	Hand-washing observed but with water alone; hand-washing facilities available; soap not readily seen at hand-washing facilities
	0	Hand-washing frequently observed at critical times; soap always/often used; hands visibly clean; hand-washing facilities seen to be regularly used
Handling: ‘Practices surrounding drinking-water collection, storage, and serving'	2	Uncovered, wide-necked container used for collection and/or storage; containers rarely washed; water served by dipping; hand-water contact regularly observed
	1	Narrow-necked collection containers; storage containers covered; hand-water contact rarely observed; storage container inaccessible to children and animals
	0	Narrow-necked collection and storage containers; water served by pouring or via tap; containers regularly washed using detergent or bleach solution
Socioeconomic: ‘Levels of education (especially hygiene) and income'	2	Few or no school facilities; rare visits by extension workers of Ministry of Health; nearest health clinic more than 4-hour walk; very basic housing; sporadic income
	1	School in/near community; moderate level of illiteracy; occasional visits by extension workers of Ministry of Health; nearest health clinic 1-2-hour walk; some paid work
	0	School in community; low level of illiteracy; opportunity for paid work and evidence of earnings; health clinic within one-hour walk; reasonable quality housing
Anthropology: ‘Cultural values and norms of the community (lifestyle factors)'	2	High health-risk occupation, e.g. re-use of animal/human wastes for agriculture; use of folk healer; childcare sharing in extended family/neighbours
	1	Moderate health risk from occupation, e.g. farming; mixed use of traditional and contemporary medicine; limited childcare sharing
	0	Little occupational health risk; use modern medicine; childcare within nuclear family

Through a combination of observation and questioning, each of the primary and secondary factors is assigned a score between zero and two. The scoring process is facilitated by the observation guide in the score sheet, which depicts conditions typical of those seen in the Honduran study communities. All or most observation points should be seen to award the score in each category, namely 0, 1, or 2. Individual observations suggested by the guide are not scored. The sum of the primary and secondary factor scores determines to which of three risk categories the household is assigned: low 0–3, medium 4–7, or high 8–10.

It would be impractical to carry out health checks to assess the second component of the DRI—‘health and immunity'. Therefore, we propose instead a surrogate indicator—nutritional status—which can be quickly and easily assessed using standard anthropometric methods. This is justified on the basis of the available evidence that suggests a strong relationship between malnutrition and immunodeficiency. More specifically, protein-energy malnutrition and micronutrient deficiencies are of particular importance to the normal functioning of the immune system (7-9). In developing countries, infants and children are one of the two main population groups (the other group being the elderly) who suffer from protein-energy malnutrition (7), and globally, more than two billion people are affected by malnutrition due to micronutrient deficiency (9). Immunoglobulin A, the most important defence against enteric pathogens, is decreased in children with protein-energy malnutrition (7).

A substantial body of evidence indicates that deficiencies in micronutrients, such as vitamin A and zinc, are linked to diarrhoeal disease and other infectious diseases in children (10-13). Bhaskaram observed that micronutrients, including vitamin A and zinc, have “…immunomodulating functions and thus influence the susceptibility of a host to infectious diseases and the course and outcome of such diseases” (8). According to the Food and Agriculture Organization, undernutrition contributes to more than 60% of mortality of children, aged less then five years (under-five mortality), caused by diarrhoea (9).

Given that children aged less than five years (under-five children) are particularly vulnerable to water-related diarrhoeal disease, the ‘health and immunity' assessment put forward in this paper makes use of a nutritional assessment for this age-group. Moreover, we propose the use of the Gomez classification of protein-energy malnutrition (Table 3), which is one of the best-known methods for evaluating nutritional status (14,15). The Gomez classification relates a child's weight to age which reflects both wasting and stunting together. Wasting is a measure of current or acute malnutrition, whereas stunting provides an indication of a child's past nutritional history and signals chronic malnutrition. The main limitation of the Gomez classification is that it cannot be used for differentiating between wasting and stunting. Nevertheless, it is both simple and straightforward to use where a rapid appraisal of nutritional status is required. Furthermore, in a study of undernutrition and childhood infections in preschool children in Sudan, a significant inverse relationship was observed between weight-for-age and subsequent infection due to diarrhoea (16) (Table 3).

**Table 3:** Gomez classification of protein-energy malnutrition

Classification of protein-energy malnutrition		% of expected weight-for-age
Normal		>90
Mild	First-degree malnutrition	76–90
Moderate	Second-degree malnutrition	61–75
Severe	Third-degree malnutrition	<60

Source: Bender DA, 1997 (17)

Having completed assessments of both ‘household water quality' and ‘health and immunity', the DRI can now be calculated using the matrix presented in Table 4. By way of example: if the ‘household water quality' assessment is ‘high' (factor score between 8 and 10), and the ‘health and immunity' assessment is ‘moderate' (second-degree malnutrition), a DRI of 4 can be read from the matrix. Depending on the criteria of the agency concerned, intervention would be called for above a DRI threshold value or priority action targeted at the highest-DRI households or communities.

**Table 4:** Disease risk index matrix used for identifying households or communities ‘most at risk' from consuming recontaminated drinking-water

Health and immunity (protein-energy malnutrition)	Household water-quality factors		
	Low	Medium	High
Normal	0	1	2
Mild	1	2	3
Moderate	2	3	4
Severe	3	4	5

### Application and goals of DRI

The primary application of the DRI is to identify households or entire communities that are ‘most at risk' from consuming recontaminated drinking-water. However, a considerable amount of information will be generated by the two appraisals which can be used in formulating action plans. For example, the ‘household water quality' appraisal of primary factors will provide useful baseline knowledge to a water and sanitation agency for designing interventions. While direct intervention of secondary factors is unlikely to be within the remit of a water and sanitation agency, it is quite feasible that action through local-level advocacy could be promoted. The results of the ‘health and immunity' appraisal could also be used through advocacy or by networking with other agencies that have a health or nutrition focus.

The DRI provides a means to set clear project goals and can be used in pre- and post-intervention appraisals. A realistic target DRI value should be set according to whether the agency is planning direct intervention or an indirect approach based on advocacy. Direct intervention would likely focus on one or more primary factor(s) to lower the ‘household water quality' score and by consequence the DRI. The selection of specific interventions would be determined by the information collected by the appraisals. The goals for advocacy or networking could be measured, for example, in terms of an increased presence of extension workers, or inclusion of communities in nutrition programmes.

The overall goal of the DRI is to provide the rationale for strategies which are designed to reduce the incidence of diarrhoeal disease. However, it is recognized that preventing the recontamination of drinking-water stored in households will only have a marginal impact on diarrhoeal disease while other transmission routes are left open. The DRI should, therefore, be seen as a tool which promotes a more integrated approach to community water supply. In this sense, the DRI offers a comprehensive planning framework for developing strategies aimed at improving water and sanitation-related hygiene. Indeed, the DRI reflects the Water Safety Plan concept advocated by WHO whereby intervention measures and preventive management practices are put in place to protect drinking-water from contamination throughout the supply chain from source to consumption (2).

### Physical and educational interventions

#### Physical measures

The WHO guidelines recommend that storage containers be designed to reduce the risk of contamination and have the following characteristics (18): (a) have a secure, tight fitting lid; (b) able to withstand rough handling without cracking; (c) easy to lift from the ground and carry back to the storage point after filling; and (d) easy to fill and clean, so that contact with hands is minimized

Although it is not explicitly stated, the above criteria imply that the storage container is also used as the collection container. The WHO guidelines also emphasize that drawing water should be possible without hands coming into contact with stored water. It is suggested that a ladle or tap is the most hygienic means to serve water from the storage container. Good hygiene practices with respect to water stored in households are also emphasized as being essential to prevent the recontamination of drinking-water. Even a well-designed storage container may not prevent recontamination if hygiene behaviour in relation to water handling is poor.

Simple physical measures, such as using lids to cover storage containers and ladles to serve drinking-water, have been promoted to prevent contamination during storage (18,19). Although these measures are proposed in conjunction with hygiene education, it is acknowledged that even basic hygiene messages relating to water storage can easily be overlooked in large projects. In a limited intervention experiment, we introduced ladles for serving water in a rural Honduran community (1). Contrary to expectation, quality of the stored water was worse following the introduction of the ladles. Presumably, the ladles were either not used exclusively for serving water or were not kept in the storage containers as instructed.

Covering storage or collection containers is another practical approach to preventing contamination by hands, airborne contaminants, or insect vectors. However, there is conflicting evidence in the literature as to whether covering the container is important with regard to preserving good quality of water and preventing transmission of diseases. Several studies have reported no significant difference in water quality in covered versus uncovered storage containers (20-23). In contrast, other research has observed an increased association between incidence of diarrhoea and uncovered storage containers (24-26).

Several studies have reported on collection/storage containers that are designed to prevent handwater contact and facilitate withdrawal of water by means of a tap or by pouring (27-30). The evidence from these studies suggests that physical measures are unable to completely prevent the deterioration of water quality on their own. It is accepted either that some deterioration of water quality will occur, or alternatively a method of treatment of household water must be considered.

**Fig. 2:**
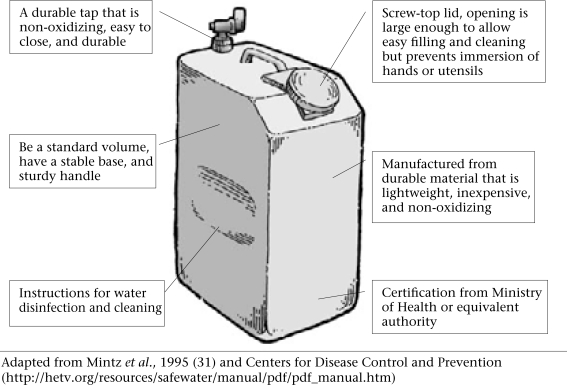
Proposed design criteria for safe water-storage container

In general, household water-treatment systems have been developed because the quality of source water is unacceptable. It has been proposed that point-of-use disinfection be combined with the use of an improved storage container (31). Studies from Bolivia, Uzbekistan, and Zambia using point-of-use disinfection and an improved container (similar to the design in Fig. 2) have reported significantly improved quality of stored water and reduced morbidity due to diarrhoea (32-35). However, it is important to note that, in these studies, the sources of water were themselves polluted. It is difficult, therefore, to make a direct comparison with studies that tested an intervention container alone and where the source water was free of faecal contamination.

Point-of-use treatment can, of course, be achieved by a number of different methods but reference to safe storage is generally absent in their description. Nevertheless, certain methods potentially offer safe storage by virtue of their design. For example, solar disinfection, widely referred to as SODIS, makes use of plastic soft-drink bottles which are filled with the water to be treated and exposed to full sunlight for a minimum of six hours (36). In suitable conditions, disinfection is achieved through a combined process of ultraviolet-A radiation and raised water temperature. Assuming that disinfected water continues to be stored in the bottles until use, then its recontamination would be unlikely. In two separate studies in a Maasai community in Kenya, the incidence of diarrhoeal disease and cholera were lower in children drinking solar-disinfected water than children from control households who kept drinking-water indoors (37,38). A comprehensive review of treatment of household water can be found elsewhere (39).

### Educational measures

Clearly, the hygienic handling of drinking-water is essential to ensure that recontamination does not occur during its collection, transportation, storage, and use. The WHO guidelines recommend that behaviours surrounding water storage, water collection, and drinking of water (drawing water from the storage container) be incorporated in hygiene-education programmes (18):

#### Water collection

- Drinking-water should be collected in clean vessels without coming into contact with hands and other materials
- Water should be transported in a covered container

#### Water storage

- Water should be stored in vessels that are covered and regularly cleaned
- Drinking-water should be stored in a separate container from other domestic water wherever possible

#### Water drinking

- Drinking-water should be taken from the storage vessel in such a way that hands, cups, or other objects cannot contaminate the water.

Similarly, Almedom describes five water and sanitation-related “clusters of hygiene behaviour”: excreta disposal, water sources, water uses, food hygiene, and domestic and environmental hygiene (40). Among the relevant features noted under ‘water sources' are methods of water collection and transportation, and for ‘water uses'-handling in the home, storage and treatment, and water use and re-use in the home. Several studies provide evidence that many of the above hygiene-related behaviours, including hand-water contact, dipping dirty utensils, dirty collection containers, and inadequate washing of storage containers, are involved in recontamination (1,27,41-45).

In summary, hygiene behaviours linked to the recontamination of drinking-water appear to have been correctly identified, and there is evidence to corroborate the health risk from drinking-water that has become contaminated during its storage. However, there is very little information in the literature that details the success or failure of educational interventions designed to prevent this problem.

Encouraging hand-washing, arguably the most basic and valuable of hygiene interventions, is much more difficult than might be imagined (44). Following a three-year hygiene-promotion programme in Burkina Faso, the number of mothers washing their hands with soap increased from 13% to 31% after cleaning a child's bottom and from 1% to 13% after using the latrine (45). Although these changes represent a significant improvement, they are still relatively modest and especially so given the direct contact with faeces. A hand-washing study in rural Bangladesh concluded that the cost of soap is a barrier to its use (46).

Only limited evidence surrounding the promotion of hand-washing as a measure to prevent recontamination of drinking-water is available, and it is largely inconclusive. For example, an intervention designed to increase the frequency and improve the efficacy of hand-washing led to a significant improvement in the quality of water stored in households (47). However, this improvement was not significant in the specific comparison of stored drinking-water. An intervention study in Guatemala City used an improved container (similar to that in Fig. 2), point-of-use disinfection, education, and soap for hand-washing to improve the microbiological quality of street-vended beverages (48). Hand-rinsed samples tested for faecal coliform counts showed that washing with soap was effective but it was observed that hands quickly became recontaminated. It was concluded that the contribution of hand-washing to the improved microbiological quality of beverage could not be quantified.

The absence of clear evidence to support a hand-washing intervention in relation to the quality of stored drinking-water should not be taken as questioning its importance in the broader context of preventing faecal-oral transmission of disease. Improvements in hand-hygiene are likely to contribute to the safety of drinking-water stored in households but are perhaps not sufficient without additional interventions. Furthermore, encouraging hand-washing before each water-handling activity would simply not be practical. It has been estimated that, if a mother washed her hands before eating, preparing food, and feeding children, this would amount to around 30 times per day (49).

The cleanliness of storage and collection containers is fundamental to ensuring that drinking-water does not become recontaminated. Results of our research in Honduras showed that thermotolerant coliforms were detectable on the inner surface of clay storage containers after simple rinsing (1). It was observed that collection containers were ‘cleaned' at the well by rubbing the hand around the inside of the container. Similar behaviour was observed in a study in Malawi, and it is probable that the intention to clean the collection container may, in fact, lead to the immediate contamination of water (30). Research data on the effectiveness of different container-cleaning regimes do not seem to be available, although the use of chlorine bleach and an abrasive agent, such as sand, gravel, or rice, is recommended in some texts (50,51).

Modifying hygiene behaviour is an enormous challenge. Current views advocate a multiple approach that draws on knowledge gained through different disciplines, including anthropology, epidemiology, marketing, communication and development studies (49). Evaluations of traditional methods of health education have not provided convincing evidence of their health impact (45,52). Nevertheless, it is suggested that health education can contribute to behaviour change through raising awareness of [waterborne] disease (51). Furthermore, education should be complemented with other techniques, such as social marketing, motivational interviewing, and community mobilization.

It is perhaps not surprising that teaching germ theory as part of a health-education programme has met with limited success. The adage ‘seeing is believing' is likely to hold true in conveying new understanding to communities with little or no formal education. It is suggested that involving consumers in water-quality testing is a valuable educational tool (53). In rural Kenya, communities were shown the visible coliform colonies following water-quality testing in wells and storage containers. This stimulated discussion of practical measures, such as regular disinfection of storage containers and wells. The idea of communities monitoring their own water systems using simple, inexpensive tests, including the presence/absence test and the hydrogen sulphide (H_2_S) test, has been studied, and field trials have been carried out in indigenous communities in Canada and Chile (54,55). Raising awareness about water quality and community empowerment are among the benefits of this educational intervention. Rijal and Fujioka suggest that the H_2_S test is suitable for use by householders to test whether solar-disinfected water is safe to drink (56).

Educational and motivational approaches to promote point-of-use disinfection and safe-water storage were compared in two peri-urban communities in Zambia (57,58). Motivational interviewing has been used in health behaviour change in the context of drug and alcohol abuse and diabetes control. The basic approach to motivational interviewing consists of careful listening, reflecting back themes that a person talks about, eliciting a person's own reasons for change, and finally helping them identify the personal resources necessary to achieve change. Ten voluntary neighbourhood health committees (hereafter referred to as committees) were trained in causes and prevention of diarrhoea, chlorine disinfection, and water storage. Five of the committees were also trained in motivational interviewing techniques. Sales of chlorine disinfectant were significantly higher among those households ‘trained' by motivational interviewing and were sustained during the eight-month study period.

In addition to participatory hygiene and sanitation transformation (PHAST) methodology, social marketing was used for promoting the use of point-of-use disinfection in rural communities in Kenya (59,60). The social marketing approach promotes the use of products for reasons other than the principal health objective of the project. For example, instead of promoting soap for hand-washing as a product to reduce diarrhoea, it is marketed on the basis that the users' hands will smell nicer. Combining community mobilization (creating demand for health interventions through participatory methods) with social marketing approaches will increase both access and demand and lead to positive behaviour change (61).

### Developing an intervention strategy

**Fig. 3:**
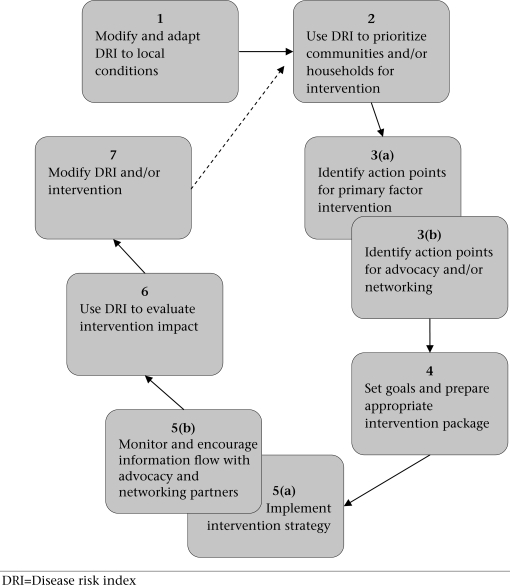
Staged development and implementation of an intervention strategy using the DRI as a planning framework

An ‘off-the-shelf' intervention that is introduced without having first carried out an appraisal of water-management practices runs the risk of being unsustainable or rejected. This is because household water-management practices are determined by many different factors, including culture, tradition, economic and aesthetic aspects. Therefore, interventions must be tailored to the particular needs of the community. Furthermore, a blanket approach to implementation might be wasteful in terms of resources if it was later shown that recipients were not among the most vulnerable sections of the population. For these reasons, the DRI has great potential as a planning framework for guiding the development of an intervention strategy. Not only will it enable the targeting of ‘most-at-risk' communities and households but also it will facilitate the development of an intervention strategy by identifying points of action specific to community or household needs. Figure 3 describes the staged development and implementation of a programme of intervention using the DRI.

The implementation of any strategy is rarely as straightforward as it may appear during desktop preparation. With regard to an intervention strate-gy that is designed to prevent or minimize the recontamination of drinking-water, there are several issues which must be resolved if the strategy is to be successful. Some of these are specific to our proposed DRI, whereas others are more general and relevant to wider issues in relation to community development programmes. The hypothetical model of the stages in an intervention strategy (Fig. 3) provides a useful starting point to discuss these issues.

The first stage of strategy development requires that the DRI is modified and adapted to local conditions. The DRI is largely the result of our research in rural Honduran communities. Consequently, the observation guide in the ‘household water quality' appraisal is based on an understanding of water-handling practices and other factors in the study communities. Although the primary and secondary factors are thought to be applicable to most community water-supply situations, location-specific experience will be needed to ‘fine-tune' the scoring mechanism. The ‘health and immunity' appraisal might also benefit from modification, and this would likely depend on the capability of the organization using the DRI. Furthermore, the DRI has yet to be field-tested, and further refinement may be necessary to make it usable by extension workers.

At the second stage, the DRI is used for identifying priority communities and households for intervention. Targeting single communities or districts for intervention is a very much accepted practice in development programmes. This can lead to a certain amount of resentment at the community level. However, the dilemma faced by an agency contemplating a household-level intervention is much greater if only the ‘most-at-risk' households are targeted. For example, how will it be perceived if only certain households are provided with the ‘special water container'? It may cause simple jealousy among neighbours; however, it might also stigmatize a family as exhibiting poor hygiene.

Detailed analysis of the DRI scores and the planning of direct and indirect interventions take place at the third stage. Assuming that the DRI is used predominantly by community water-supply organizations, it can be expected that their main capability will relate to the primary factors affecting the quality of household water. However, it is often the case that such organizations are involved in advocacy and could also plan for indirect action to influence secondary factors. Furthermore, partnerships and consortia are common, and action aimed at improving health and immunity is also conceivable. At this stage, an outline intervention strategy is drafted indicating whether the identified action points are to be addressed by direct intervention, advocacy, or through networking partnerships.

In the fourth stage, setting goals and selecting appropriate interventions are closely interlinked. It is important to be realistic about what can be achieved in terms of lowering the DRI and in what timeframe. Where external funding agencies are involved, they will need to be convinced that the proposed interventions are cost-effective. Individual interventions will have a varying impact on household water-quality factors. Some interventions may produce an immediate result, whereas others will require a much greater time to produce the desired effect. For example, introducing a specially-designed container that prevents hand-water contact will be immediately effective. In contrast, interventions aimed at achieving hygiene-behaviour change are widely accepted to be a long-term process. Furthermore, step improvements in the DRI can be expected because, despite the careful selection of interventions, there may still be unforeseen barriers to their uptake.

There are several issues to consider with regard to selecting appropriate interventions, not least of which is the issue of cost. Most physical interventions will have to be purchased, extension workers are needed for educational interventions, and modified household water management costs time and effort. How these costs are shared impinges upon both uptake and sustainability of the interventions. With respect to physical interventions, such as special containers, the organization must decide whether to donate, subsidise, or sell at full cost.

The particular circumstances in which an intervention is introduced can have a significant bearing on their uptake and users' willingness to share costs. For example, special containers, similar to the design shown in Figure 2, introduced in poor areas of Karachi were initially well-received. However, with the onset of hot weather, the containers were largely abandoned because of the difficulty of adding ice to the special container and its lack of insulating properties (62). A relatively-high adoption rate for point-of-use disinfection in rural Kenya was thought partly due to the concerns of communities about diarrhoeal disease (59). Point-of-use disinfection was also promoted in Madagascar following an outbreak of cholera (61). However, demand for bottled disinfectant was related to peaks in the epidemic of cholera, suggesting that it was perceived as necessary only for preventing cholera. Furthermore, questions were raised concerning the sustainability of supplying disinfectant because full cost-recovery was not achieved.

Point-of-use disinfectants tend to be chlorine-based, and doubts are often voiced about the acceptability of chlorine to users because of taste and odour problems. There are also questions surrounding the affordability of bottled disinfectant and the issue of distribution to isolated rural communities. As an alternative, the concept of SODIS has distinct advantages over chlorine disinfectants with respect to cost and taste. However, it is highly dependent on climatic conditions and may not be practical for large households in which a correspondingly large number of bottles would be needed.

Stage five represents the implementation phase of the strategy. Distinct approaches will suit the various interventions being used. In the case of interventions directed at the primary factors of quality of household water, participative approaches are recommended as most likely to result in a sustainable uptake of the interventions. Indirect intervention through advocacy and networking will take a different approach. Advocacy requires diplomatic persuasion aimed at organizations and individuals that are in a position of influence. Networking, on the other hand, could involve memoranda of agreement among organizations, sharing of information, and, perhaps, mutual commitment to provide input to communities where partner organizations operate. All intervention approaches should be continuously monitored so that the strategy can be refined and improved even during its implementation.

The DRI is used again at stage six to carry out an evaluation of the impact of the intervention strategy. Aside from the hope for reduction in the DRI, a broad view should be taken during this evaluation as it is possible that water and sanitation-related practices may have changed, although they were not the direct focus of intervention. This can happen because of the strong interrelationship among water, sanitation and hygiene practices. Even a focused intervention is likely to lead to raised awareness of other links in this ‘chain' of sanitary practices.

Finally, stage seven is a suitable point at which to review both DRI and interventions used in the strategy. The experience gained with the DRI in prioritizing communities (stage two), and then evaluating the impact of the intervention strategy (stage six), may indicate that the DRI needs further modification to improve its sensitivity to the local conditions. The interventions might also benefit from modification, although may equally they be substituted for alternatives if they have not led to the desired result. At this point, the cycle begins again, starting at stage two, unless, of course, no further reduction in the DRI is considered to be necessary.

## CONCLUSIONS

Designing appropriate interventions to minimize the recontamination of drinking-water is important because of limited resources, which should be directed at the most vulnerable populations. The DRI proposed in this paper offers a means of identifying those ‘most at risk' and, moreover, a planning framework on which to develop an integrated strategy to address water and sanitation-related hygiene. We suggest that the DRI could be easily incorporated into the existing community water-supply strategy and would pose a minimal burden in terms of needing new organizational learning.

Preventing or minimizing recontamination of drinking-water does not necessarily require new intervention measures, as there are numerous methods which are capable of achieving this goal. The challenge for community water-supply programmes is to make use of interventions that are appropriate to the needs and preferences of the target users. Here also, the DRI, more specifically the ‘household water quality' appraisal, can be used for collecting information about water-management practices that will facilitate the selection of appropriate interventions. However, there remain other issues that will have to be resolved, including those relating to sharing of cost where physical interventions are introduced and whether entire communities or individual households are the focus for intervention.

The DRI still needs to be field-tested to determine its usefulness in community water-supply programmes, and it will undoubtedly require modification and adaptation to the specific conditions in which it is to be used. However, we envisage that field-testing should be relatively straightforward given that the DRI is essentially a tool designed to make use of the existing interventions and appraisal methods. The criteria on which it will be judged are two-fold. First, it should ensure that the quality of drinking-water is maintained between the points of collection and consumption. And second, it should contribute to a holistic approach to community water supply.
